# A Fast MEANSHIFT Algorithm-Based Target Tracking System

**DOI:** 10.3390/s120608218

**Published:** 2012-06-13

**Authors:** Jian Sun

**Affiliations:** 1 State Key Laboratory for Strength & Vibration, School of Aerospace, Xi'an Jiaotong University, Xi'an 710049, China; E-Mail: sunjian10@xjtu.edu.cn; Tel.: +86-029-8258-2547; 2 State Key Laboratory of Precision Measurement Technology and Instruments, Tsinghua University, Beijing 100084, China; 3 College of Astronautics, Northwest Polytechnical University, Xi'an 710072, China

**Keywords:** target tracking system, moving object tracking, fast Mean-Shift algorithm

## Abstract

Tracking moving targets in complex scenes using an active video camera is a challenging task. Tracking accuracy and efficiency are two key yet generally incompatible aspects of a Target Tracking System (TTS). A compromise scheme will be studied in this paper. A fast mean-shift-based Target Tracking scheme is designed and realized, which is robust to partial occlusion and changes in object appearance. The physical simulation shows that the image signal processing speed is >50 frame/s.

## 1. Introduction

Visual tracking plays an important role in various computer vision applications, such as surveillance [1,2], firing systems [3], vehicle navigation [4] and missile guidance [5]. Target tracking using an active video camera is a challenging task mainly due to three reasons [6–8]: (1) the tracking system should have good robustness to the targets' pose variation and occlusion; (2) tracking requires properly dealing with video camera motion through suitable estimation and compensation techniques; (3) most applications would introduce some real-time constraints, which require tracking techniques to reduce the computational time [5].

Target tracking, according to its properties, can be mainly divided into two types: feature- and optical flow-based approaches. Optical flow is the vector field which describes how the image changes with time [9]. The amplitude and direction of the optical flow vector of each pixel is usually computed by the Lucak-Kande algorithm. Shi and Tomasi [10] also proposed the well-known Shi-Tomasi-Kanade (STK) tracker which iteratively computes the translation of a region centered on an interest point [9]. However, optical flow computation is too complicated to meet real-time requirements, and it is sensitive to illumination changes and noises, which limit its practical application.

Feature-based algorithms were originally developed for tracking a small number of salient features in an image sequence. These features include: color, grain, contour and some detection operators such as invariant feature transform (SIFT) [9] or histogram of oriented gradient (HOG) [11]. Feature-based algorithms involve the extraction of regions of interest in the images and then location of the target in individual images of the sequence. Typical feature-based tracking algorithms are: multiple hypothesis tracking (MHT) [12], Template Matching (TM) [13–16], Mean-Shift (MS) [17–19], Kalman filtering (KF) [20] and particle filter (PF) [21,22].

The TM is a simple and popular technique in target tracking, which is widely used in civilian and military automatic target recognition systems. Given an input and a template image, the matching algorithm finds the partial image that most closely matches the template image in terms of some specific criterion, such as the Euclidean distance or cross correlation. The conventional template matching methods consume a large amount of computational time. A number of techniques have been investigated with the intent of speeding up the template matching, and have given perfect results [14,15]. However, the TM does not achieve robust performance in complex scenes, especially in the case of clutter and occlusion [3].

The Kalman filter and particle filter are used to estimate target location in the next frame, which has also been extensively studied. Comparing to the Kalman filter, the particle filter has a more robust performance in the case of nonlinear and non-Gaussian problems due to the simulated posterior distribution. Many efforts have been carried out to speed up the particle filter. Martinez-del-Rincon *et al.* [21] proposed a new particle filter algorithm based on two sampling techniques, which improves substantially the efficiency of the filter. Sullivan et al. [23] proposed layered sampling using multiscale processing of images. It turns out that these solutions significantly reduce the computational costs, but in-depth efforts are desirable for better efficiency.

In image sequences, the target appearances have a strong correlation. Among all appearance based tracking models, there is one popular subset called “subspace model”. Black [24] used a set of orthogonal vectors to describe the target image. Principal Component Analysis (PCA) and other classic dimensionality reduction methods provide an effective tool to compute the set of orthogonal vectors. Levy and Linden-Baum [25] presented a novel incremental PCA algorithm (Sequential Kathunen-Loeve, SKL) to update the eigen-basis when new data is available with greatly reduced computation and memory requirements. Lin applied Fisher linear discriminant analysis in subspace tracking to take background into account [26], however, it cannot perform well in case of non-Gaussian distribution.

The MS based tracker has very good robustness to the variation of translation, rotation and scale. The MS algorithm is a nonparametric density gradient estimation approach to local mode seeking and it was originally invented for data clustering. Comaniciu [18] was the first to develop its application in target tracking. The tracker needs a target model to be able to track. The target model is obtained from the color histogram of the moving object. The target candidate is obtained in the same way at a location specified by the MS algorithm. The similarity measure between the target candidate and the target model is computed using the Bhattacharya coefficient.

One of MS's drawbacks is that it often converges slowly. To the best of our knowledge, few attempts have been made to speed up the convergence of MS. The k_d_-tree can be used to reduce the large number of nearest-neighbor queries. Although a dramatic decrease in the computational time is achieved for high-dimensional clustering, these techniques are not attractive for relatively low-dimensional problems such as visual tracking. Cheng [27] showed that mean shift is gradient ascent with an adaptive step size, but the theory behind the step sizes remains unclear.

The innovative work in this paper is to propose a novel fast robust tracking algorithm combining the MS with the template match (TM), which is a balanced scheme between robustness and real-time performance. A fast MS-based target tracking scheme is designed and implemented, which has a good robustness to target pose variation and partial occlusion. The hardware-in-loop simulation shows that the image signal processing speed is >50 frame/s.

The paper is organized as follows: the target tracking system description is described in Section 2, the hardware composition is presented in Section 3, the software structure and algorithm are described in details in Section 4, and, finally, Section 5 reports tests and results, and Section 6 describes the future works.

## 2. System Description

**Figure 1.**
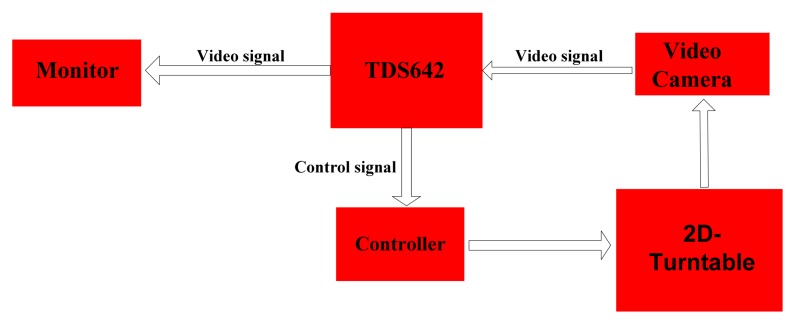
The target tracking system structure chart.

As shown in Figure 1, the target tracking system in this paper mainly has the following parts: video camera, signal processing module, monitor and 2D-turntable. In order to meet some practical application requirements the TTS must to have the following two performance features:

1. Robustness. In a complex background, most of the applications require the tracker to be robust to partial occlusion, clutter and changes in object appearance.
2. Real-time performance. TTS needs to complete the image signal pre-processing, tracking and predicting target location, control 2D-turntable and other computational tasks which requires that the image processing speed should be >25 frames/s, and for some special applications processing speeds need to be >50 frame/s.

**Figure 2.**
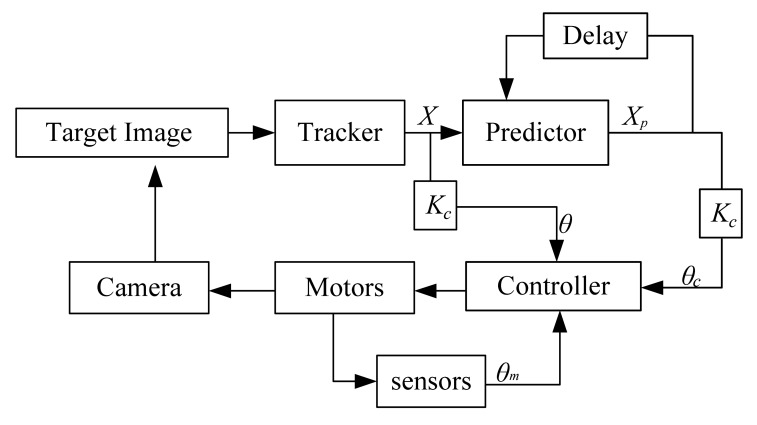
Signal flow diagram of a typical target tracking system.

The signal flow diagram of a typical target tracking system is shown in Figure 2. The TTS obtains the target image by a video camera. Through a tracker, the target location *X* in the current image is obtained and sent to the predictor to predict the target location *X_p_* in the next frame. The predicted result *θ_c_*, the desired angle *θ* and the feedback angle *θ_m_* are used to control the 2D-turntable.

## 3. Hardware Composition

### 3.1. Signal Processing Module

The video signal processer used in this paper is the TDS642EVM multi-channel real-time image processing platform produced by the TI Company. Its main performance features are listed in Table 1.

**Figure 3.**
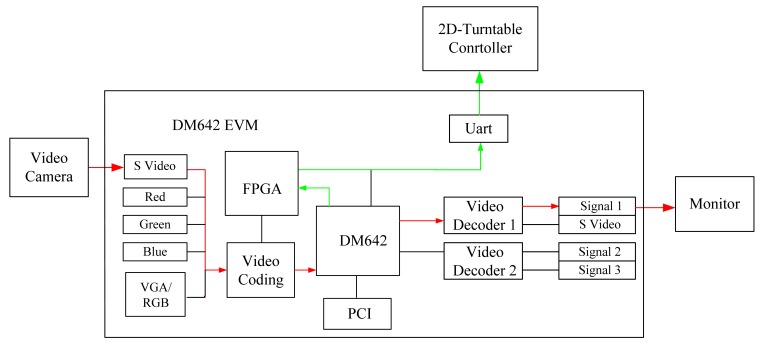
The structure of TDS642EVM.

The structure of the TDS642EVM is shown in Figure 3. The red line denotes video signal flow; the green line denotes control signal flow.

### 3.2. Video Camera and 2D Turntable

**Figure 4.**
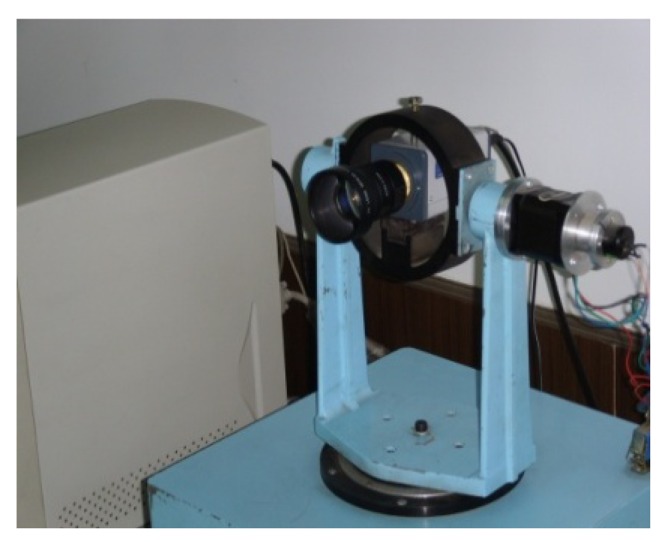
2D turntable and video camera.

The pitch and yaw axis of 2D-turntable (as shown in Figure 4) are linked with the output shaft of the stepping motor, respectively. The control of the 2D-turntable is realized by controlling the two stepper motors. The turntable controller obtains control instructions from TDS642EVM by a UART, and generates the pulse signal to drive the stepping motor. The rotation angle of the turntable measured by a potentiometer is used as the feedback for the closed-loop control system. The performance characterstics of the 2D turntable are given in Table 2.

## 4. Software Structure and Algorithm

**Figure 5.**
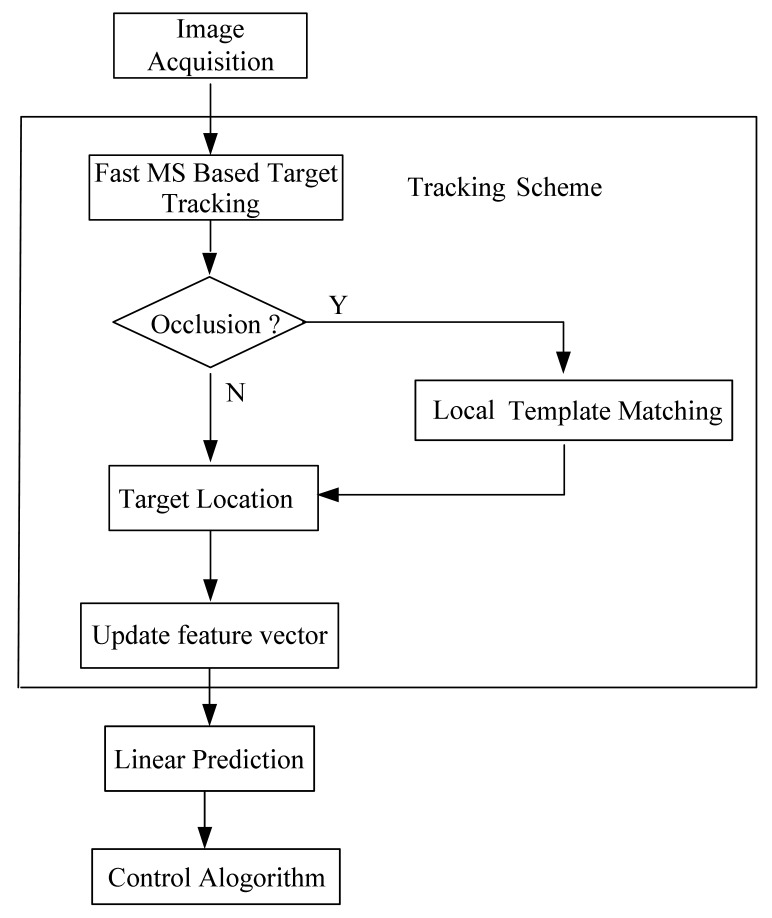
The structure of the TTS software structure.

The structure of the TTS software is shown in Figure 5. The TTS software mainly includes the following two parts: image tracking algorithm, the target prediction algorithm.

1. The tracking algorithm is to identify the location of the target in the current image. A fast robust MS-based target tracking algorithm is presented.
2. The target prediction algorithm is to predict the location of the target in the next image though the sequence image. There are many algorithms that can achieve the prediction goal such as Kalman filter, particle filter and linear prediction method. Although the Kalman filter and particle filter [20,21] have obtained good results, these two algorithms are both inefficient. In this paper we use a linear prediction method to implement the target location prediction.

### 4.1. Fast MS Tracking Scheme

#### 4.1.1. Mean-Shift Basis [19]

Kernel density estimation is a nonparametric method that extracts information about the underlying structure of a data set when no appropriate parametric model is available. Given *n* data points *x_i_*, *i* = 1 *n*, in the *d* dimensional space **R**^d×d^, the kernel density estimation at the location *x* can be computed by:

(1) $${\hat{f}}_{k}\left(x\right)=\frac{c_{k}}{nh^{d}}\sum_{i=1}^{n}k\left({\left\|\frac{x-x_{i}}{h}\right\|}^{2}\right)$$

where *k*(·) is the profile the kernel function *K*(·) and *c_k_* is a normalization constant. The optimization procedure of seeking the local modes is solved by setting the gradient equal to zero. Thus, we can derive the following equation:

(2) $$\begin{array}{l} m_{G}\left(x\right)=\frac{\overset{n}{\underset{i=1}{\text{∑}}}x_{i}g\left({\left\|\frac{x-x_{i}}{h}\right\|}^{2}\right)}{\overset{n}{\underset{i=1}{\text{∑}}}g\left({\left\|\frac{x-x_{i}}{h}\right\|}^{2}\right)}-x \end{array}$$

where *g*(*x*) = −*k′*(*x*), *m_G_*(*x*) is the MS vector.

#### 4.1.2. Target Description and Distance Metric

According to the classical MS tracking algorithm [19], we can compute the target and candidate target feature vectors as follows:

Target feature vectors:

(3) $${\hat{q}}_{u}=C\sum_{i=1}^{n}k\left({\left\|x_{i}\right\|}^{2}\right)\delta \left[b\left(x_{i}\right)-u\right],u=1\cdots m$$

Candidate target feature vectors:

(4) $${\hat{p}}_{u}\left(y\right)=C_{h}\sum_{i=1}^{n_{h}}k\left({\left\|\frac{y-x_{i}}{h}\right\|}^{2}\right)\delta \left[b\left(x_{i}\right)-u\right],u=1\cdots m$$

where *δ* is the Kronecker delta function, *b*(*x_i_*) is the quantified number of the pixels value in the quantitative feature space, *C*, C*_h_* are the normalization constants.

The similarity function defines a distance between target model and candidates. To accommodate comparisons among various targets, this distance should have a metric structure. We define the distance between two discrete distributions as:

(5) $$d\left(y\right)=\sqrt{1-\rho \left[\overset{⌢}{p}\left(y\right),\overset{⌢}{q}\right]}$$

(6) $$\overset{⌢}{\rho }\left(y\right)=\rho \left[\overset{⌢}{p}\left(y\right),\overset{⌢}{q}\right]=\sum_{u=1}^{m}\sqrt{{\overset{⌢}{p}}_{u}\left(y\right){\overset{⌢}{q}}_{u}}$$

where *ρˆ*(*y*) named Bhattacharyya coefficient.

#### 4.1.3. Tracking Algorithm

To find the location of the target in the current frame, the distance (5) of a function of *y* should be minimized. The tracking starts from the location of the target in the previous frame and searches in the neighborhood. Minimizing the distance (5) is equivalent to maximizing the Bhattacharyya coefficient *ρˆ*(*y*).

Thus, the probabilities {*pˆ_u_*(*y_0_*)}_u=1,…_*_m_* of the target candidate at location *y*_0_ in the current frame must be computed first. Using Taylor expansion around the values *pˆ_u_*(*y_0_*), the linear approximation of the Bhattacharyya coefficient (6) is obtained after some manipulations as:

(7) $$\rho \left[\overset{⌢}{p}\left(y\right),\overset{⌢}{q}\right]\approx \frac{1}{2}\sum_{u=1}^{m}\sqrt{{\overset{⌢}{p}}_{u}\left({\overset{⌢}{y}}_{0}\right){\overset{⌢}{q}}_{u}}+\frac{1}{2}\sum_{u=1}^{m}{\overset{⌢}{p}}_{u}\left(y\right)\sqrt{\frac{{\overset{⌢}{q}}_{u}}{{\overset{⌢}{p}}_{u}\left({\overset{⌢}{y}}_{0}\right)}}$$

This approximation is satisfactory when the candidate {*pˆ_u_*(*y*)}*_u_*_=1,…_*_m_* and the initial {*pˆ_u_*(*yˆ*_1_)}*_u_*_=1,…_*_m_* are little difference. In general, for adjacent two frames this assumption is reasonable. Thus we have:

(8) $$\rho \left[\overset{⌢}{p}\left(y\right),\overset{⌢}{q}\right]\approx \frac{1}{2}\sum_{u=1}^{m}\sqrt{{\overset{⌢}{p}}_{u}\left({\overset{⌢}{y}}_{1}\right){\overset{⌢}{q}}_{u}}+\frac{C_{h}}{2}\sum_{i=1}^{n_{h}}w_{i}k^{\prime }\left({\left\|\frac{y-x_{i}}{h}\right\|}^{2}\right)$$

In which:

(9) $$w_{i}=\sum_{u=1}^{m}\delta \left[b\left(x_{i}\right)-u\right]\sqrt{\frac{{\overset{⌢}{q}}_{u}}{{\overset{⌢}{p}}_{u}\left({\overset{⌢}{y}}_{1}\right)}}$$

In this way, minimizing *d*(*y*) becomes to maximize the second of Equation (8), which denotes the kernel density estimation computed by using *k*(*x*) at the *y* in current frame. In this process, the kernel shifts from the current location *y* to the new location *y*_1_. Thus we can use the MS procedure to find the great density estimation value in the neighborhood:

(10) $$\begin{array}{l} y_{1}=\frac{\sum_{i=1}^{n_{h}}x_{i}w_{i}g\left({\left\|\frac{y-x_{i}}{h}\right\|}^{2}\right)}{\sum_{i=1}^{n_{h}}w_{i}g\left({\left\|\frac{y-x_{i}}{h}\right\|}^{2}\right)} \end{array}$$

The general MS algorithm steps are as follows [19]:

Given: the target model {*q_u_*}_u=1,…,m_ at *y_0_* in the previous frame, *y*_1_ is the new location of spot. Then the flow of MS algorithm is:

Set the spot with a feature vector {*q_u_*}*_u_*_=1,…,_*_m_*, at *y*_0_ in the previous frame.

1. Compute the feature vector of candidate spot {*p̑_u_*(*y̑*_0_)}*_u_*_=1,…,_*_m_*, and evaluate Bhattacharyya coefficient $\rho \left[\overset{⌢}{p}\left({\overset{⌢}{y}}_{0}\right),\overset{⌢}{q}\right]=\sum_{u=1}^{m}\sqrt{{\overset{⌢}{p}}_{u}\left({\hat{y}}_{0}\right){\overset{⌢}{q}}_{u}}$
2. Derive {*w_i_*}*_i_*_=_*_1_*_…_*_m_* with Equation (9).
3. Find the new location of spot with Equation (10)
4. Compute {*p̑_u_*(*y̑*_1_)}*_u_*_=1,…_*_m_* and evaluate $\rho \left[\overset{⌢}{p}\left({\overset{⌢}{y}}_{1}\right),\overset{⌢}{q}\right]=\sum_{u=1}^{m}\sqrt{{\overset{⌢}{p}}_{u}\left({\hat{y}}_{1}\right){\overset{⌢}{q}}_{u}}$.
5. While *ρ*[*p̑*(*y̑_1_*), *q̑*]< *ρ*[*p̑*(*y̑_0_*), *q̑*]
  Do ${\overset{⌢}{y}}_{1}\leftarrow \frac{1}{2}\left({\overset{⌢}{y}}_{0}+{\overset{⌢}{y}}_{1}\right)$
  Evaluate ρ[*p̑*(*y̑*_1_), *q̑*]
6. If ║ *y̑*_1_ − *y̑*_0_ ║< *ε* stop iteration.
  Else *y̑*_0_ ← *y̑*_1_ jump to 2

#### 4.1.4. Fast Tracking Algorithm

References [27,28] show that MS is actually a bound maximization. One step of the MS iteration finds the exact maximum of the lower bound of the objective function. The existing literatures [21,29–33] also show that MS is a gradient ascent algorithm with adaptive step size. Hence, its convergence rate is better than conventional fixed-step gradient algorithms and no step-size parameters need to be tuned [17]. From the viewpoint of bound optimization, the learning rate can be over-relaxed to make its convergence faster.

From another point of view, bound optimization methods always adopt conservative bounds in order to guarantee increasing the cost function value at each iteration [17]. A lot of work has been done to speed up bound optimization methods. In [17,29], it was shown that by over-relaxing the step size, acceleration can be achieved. Supposing M_G_ is the MS shift vector, and then the over-relaxed bound optimization iteration is given by:

(11) $$y^{\left(k+1\right)}=y^{\left(k\right)}+\alpha \cdot {\text{M}}_{\text{G}}$$

Apparently when the *α* = 1, over-relaxed optimization reduces to the standard MS algorithm. It is easily found that when *α* > 1 acceleration is realized, but for a fixed *α*, no convergence is guaranteed and it is hard to get the optimal *α* [17]. References [17,31] prove that in the case of general bound optimization model, convergence can be secured using the over-relaxed bound optimization iteration when the candidate are close to a local maximum and 0 < *α* < 2. Based on this proposition, an adaptive over-relaxed bound optimization is readily available: *α* can be adjusted by evaluating the cost function. When the cost function becomes worse for some *α* > 1, then *α* has been set too large and needs to be reduced. By setting *α* = 1 immediately, convergence can be achieved. In this paper, we presented the accelerated MS algorithm as follows:

1. Initialization:
  Set the iteration index *k* = 1, and the step parameter *β* > 1, *α* = 1.
2. Iterate until convergence condition is met:
3. Compute *yˆ_i_*_+1_ with Equation (13). And the MS vector m_G_(*yˆ_i_*_+1_)= *yˆ_i_*_+1_*− yˆ_i_*.
4. *y_i_*_+1_ = *y_i_*+ α m_G_(*yˆ_i_*_+1_)
5. If *ρ*(*y_i_*_+1_)*> ρ*(*y_i_*)
6. Accept *y_i_*_+1_ and *α* = 1, *α* = *β·α*.
7. Else reject *y_i_*_+1_, and *y_i_*_+1_ = *yˆ_i_*_+1_, *α* = 1.
8. Set *k* = *k +* 1, start a new iteration
9. If m_G_(y*_i_*_+1_)<*ε* stop iteration.

#### 4.1.5. Case Study

**Figure 6.**
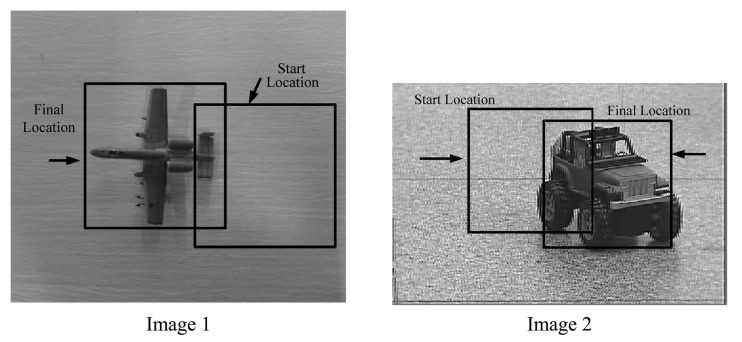
Two images for fast MS *versus* the standard MS.

We compare the performance of the accelerated MS algorithm to the standard MS algorithm on real images (as shown in Figure 6). In the experiments, all codes run on the EVM642 mentioned in Section 3. We repeat all the tests 10 times and the average CPU time is reported in Table 3. From the test results we can conclude that the Fast MS is at least three times faster than the standard MS.

#### 4.1.6. Occlusion Issue

The occlusion issue is a technical challenge in the image tracking field. Many methods have been proposed to solve this problem. In this paper, the Bhattacharyya coefficient is used to determine whether the target is in occlusion or lost. Setting thresholds T1, T2, if T1 < Bhattacharyya coefficients < T2, the target is considered to be occluded, if Bhattacharyya coefficients < T1, the target is considered to be lost. In addition, by the effects of the environment illumination and the target appearance changes, the Bhattacharyya coefficient of the target candidate is, in general, the local maximum rather than the global maximum. When the target is in occlusion, the distance between the local maximum and the global maximum would increase, so some special method needs to be implemented to improve the tracking robustness. The Local Template Matching (LTM) method is used in this article to solve this problem. Template Matching (LM) is an existing algorithm, and, usually, it is a global template matching technique. In this paper template matching is implemented in the region of the candidate target, so here it is called Local Template Matching.

**Figure 7.**
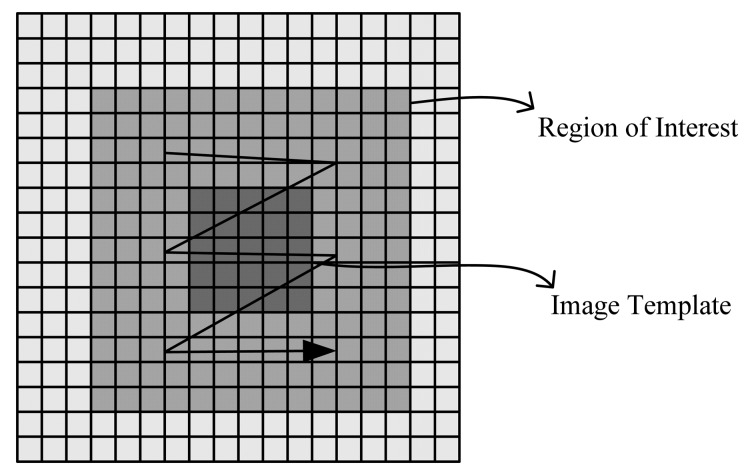
The template matching algorithm.

The final location (*x*, *y*) of the target is computed over a region of interest (ROI) surrounding the candidate location derived from the fast MS as shown in Figure 7. The LTM algorithm is as follows:

(12) $$D\left(x,y\right)=\sum_{u=1}^{M}\sum_{v=1}^{M}R\left(u+x,v+y\right)-S\left(x,y\right)$$

where *S*(*x*, *y*) is the pixel value at (*x*, *y*) in template image, *R*(*u*+*x*, *v*+*y*) is the pixel value at (*u*+*x*, *v*+*y*) in the search area. (*u*, *v*) is the candidate location derived from the fast MS. *D*(*x*, *y*) is the distance in the feature space, and a smaller value shows a higher correlation. Then the minimum distance *D*_Min_(*x*, *y*) and the corresponding location (*x*, *y*) are determined.

### 4.2. Target Prediction Algorithm

In order to improve the TTS response speed it is necessary to use the prediction method in the tracking scheme. Compared to the Kalman filter and particle filter, the linear prediction algorithm is less complex and offers moderate performance. In this paper we use the linear prediction method to get the predicted angular position of the target.

A simple method to estimate the location of the target in the image can be formulated by the following equation:

(13) $$\left({\hat{x}}_{t+1},{\hat{y}}_{t+1}\right)=\left(x_{t}+\Delta {\hat{x}}_{t+1},y_{t}+\Delta {\hat{y}}_{t+1}\right)\approx \left(x_{t}+\Delta x_{t},y_{t}+\Delta y_{t}\right)$$

where (*xˆ_t_*_+1_, *yˆ_t_*_+1_) represents the estimated location of the target, (Δ *xˆ_t_*_+1_, Δ *yˆ_t_*_+1_) represents the estimated shift vector from *t* to *t* + 1, (Δ *x_t_*, Δ *y_t_*) represents the real shift vector from *t* − 1 to *t*. This method assumes that the shift vector of velocity of the target is unchanged in a short time.

Another advanced algorithm which formulates the shift vector (Δ*xˆ_t_*_+1_, Δ*yˆ_t_*_+1_) as a linear combination of the shift vectors: {(Δ*x_t_*_−_*_k_*, Δ*y_t_*_−_*_k_*), (Δ*x_t_*_−_*_k_*_+1_, Δ*y_t_*_−_*_k_*_+1_),…, (Δ*x_t_*, Δ*y_t_*)}. Then the following equation is:

(14) $$\Delta {\hat{x}}_{t+1}=a_{k}\cdot \Delta x_{t-k}+a_{k-1}\cdot \Delta x_{t-k+1}+\cdots +a_{0}\cdot \Delta x_{t}$$

(15) $$\Delta {\hat{y}}_{t+1}=b_{k}\cdot \Delta y_{t-k}+b_{k-1}\cdot \Delta y_{t-k+1}+\cdots +b_{0}\cdot \Delta y_{t}$$

where *a_k_*,…,*a*_0_,*b_k_*,…,*b*_0_ is a group of fix coefficients which are set offline.

The 2D-Turntable's pitch and yaw angular deviation can be obtained by the following formula:

(16) $$\Delta \theta =\left(\begin{array}{c} \Delta \theta_{x}\\ \Delta \theta_{y} \end{array}\right)=\left(\begin{array}{c} k_{1}\\ k_{2} \end{array}\right)\left(\begin{array}{c} \Delta {\hat{x}}_{t+1}\\ \Delta {\hat{y}}_{t+1} \end{array}\right)$$

where Δ*θ_x_* and Δ*θ_y_* is respectively the pitch and yaw angular deviation.

**Figure 8.**
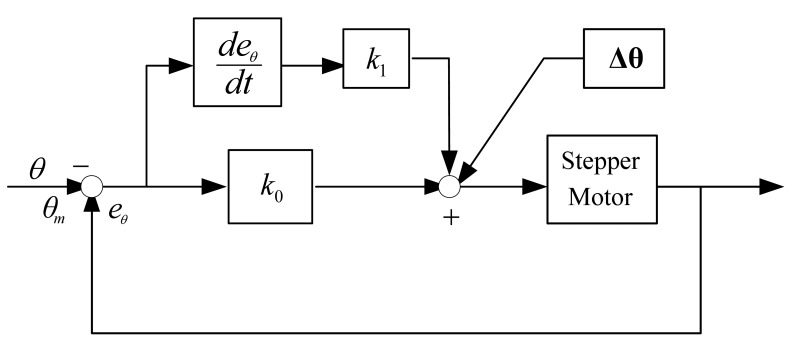
The scheme of the feed forward compensation-based PD controller.

A reliable PD controller is used for the tracking system, and the angular deviation **Δθ** obtained from linear prediction is used as feed forward compensation, then the final control algorithm is:

(17) $$\text{u}={\text{k}}_{0}*(\theta -\theta_{m})+{\text{k}}_{1}*\frac{d{\text{e}}_{\theta }}{\mathrm{dt}t}+\Delta \theta$$

where **θ** represents the angles of the instruction, **θ***_m_* represents the angle of the feedback. The scheme of the feed-forward compensation based PD controller is shown in Figure 8.

(18) $${\text{e}}_{\theta }=\theta -\theta_{m}$$

## 5. Experiments Section

### 5.1. Parameter Setting

The kernel function has an important influence on the experimental results. In this paper the Epanechnikove kernel profile is used as:

(19) $$K_{E}(x)=\left\{ \begin{array}{l} \begin{array}{cc} C_{k}(1-\|\frac{x}{z}{\|}^{2}) & \|\frac{x}{z}{\|}^{2}\leq 1 \end{array}\\ 0\;\|\frac{x}{z}{\|}^{2}>1 \end{array}\right.$$

where *z* = 128 is the bandwidth of MS tracking algorithm which is decided by the size of the target. *x* actually represents the distance between the effective pixels and the center of the tracking region.

The quantization function *b* is:

(20) $$b(x)=\{\begin{array}{cc} 1 & 0<x<60\\ 2 & 110\leq x\leq 160\\ 3 & 160<x<210\\ 4 & 210\leq x<255 \end{array}$$

Region of interest (ROI) is 20 × 20.

### 5.2. Experiments Results

**Figure 9.**
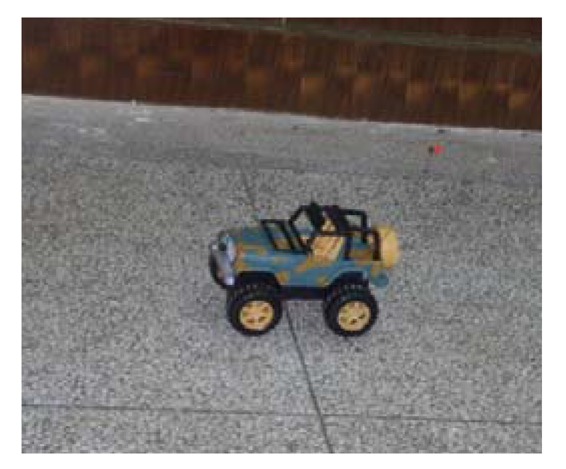
The wireless remote car.

**Figure 10.**
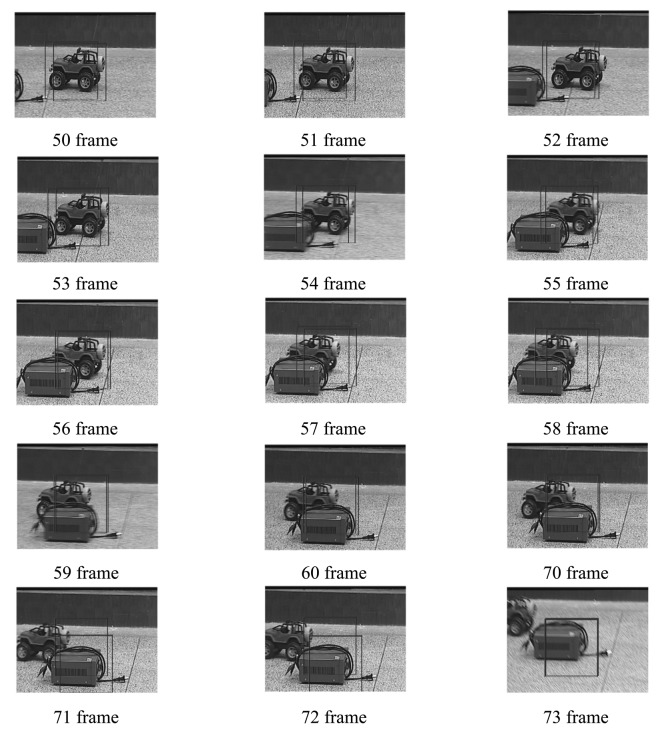
Tracking with the traditional MS.

**Figure 11.**
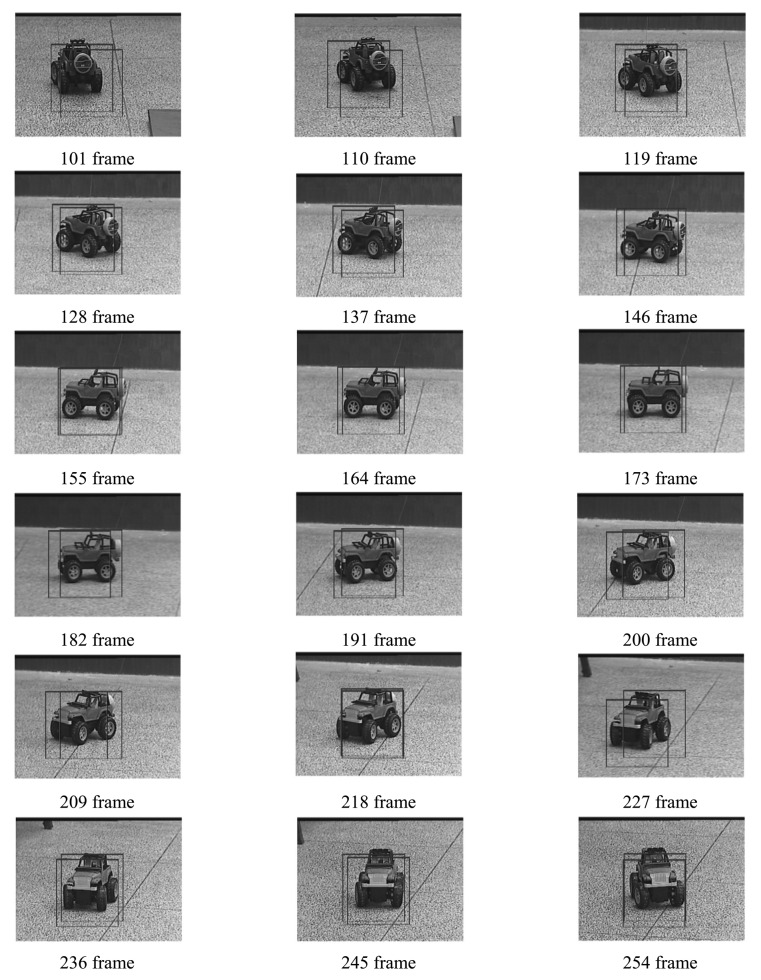
Tracking with proposed method in case of poses variation.

**Figure 12.**
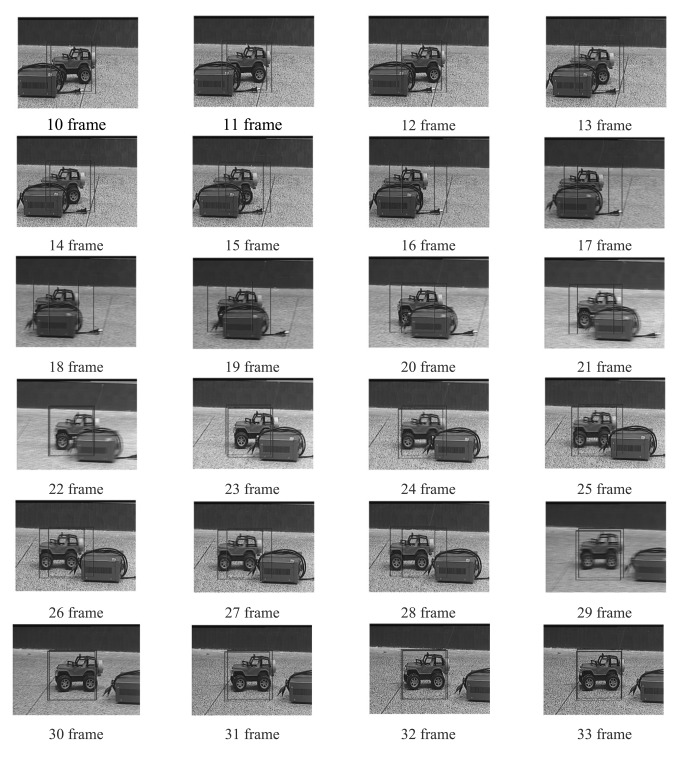
Tracking with proposed method in case of occlusion.

**Figure 13.**
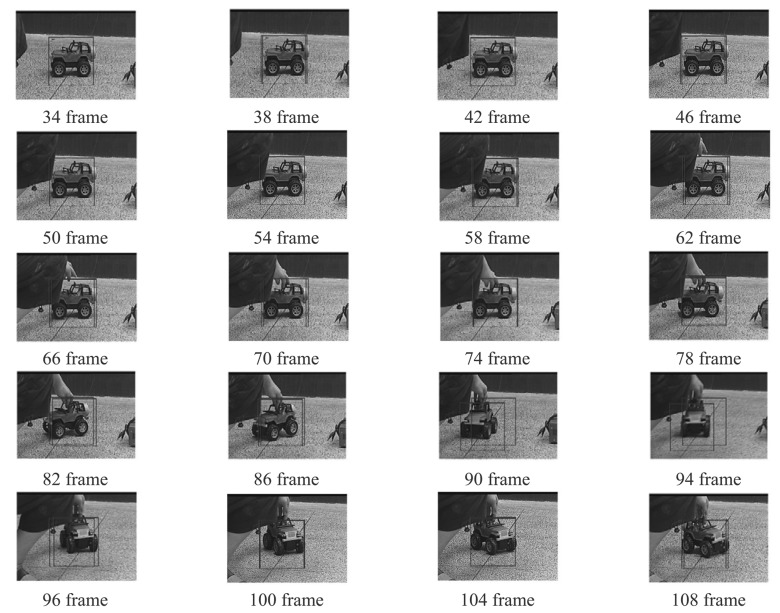
Tracking with proposed method in case of poses variation under complex scene.

Four experiments have been implemented to test the above target tracking scheme. A wireless remote control car (as shown in Figure 9) has been used to simulate a moving target. The experiments include four cases: in case of tracking with the traditional MS (as shown in Figure 10), tracking in case of poses variation with the proposed method (as shown in Figure 11), tracking in case of partial occlusion with the proposed method (as shown in Figure 12), tracking in the case of poses variation in a complex scene with the proposed method (as shown in Figure 13).

From the following tracking image sequence, we can find two rectangular boxes. One represents the center of the optical system; the other represents the target location in the current image. The distance between the two rectangular boxes are used as errors to control the 2D-turntable. When the target is in stop condition, the two rectangular boxes should overlap.

From the following experiments results, we can conclude that the TTS designed in this paper has good robustness to the target pose variation and occlusion. The system totally processes an image in 18.21 ms, in which the fast MS consuming 14.6 ms, TM consumes 1.83 ms, other algorithms consume 1.78 ms. The Target Tracking Scheme time-consuming statistical table is as shown in Table 4. The final image processing speed is >50 frame/s. The experiment results indicate our approach to tracking a moving target is fast and robust. However, this proposed algorithm needs to be comprehensively evaluated in a wider database. Although the tracking results are promising in certain situations, further development and more evaluation is anticipated in severe image clutter and occlusion situations.

## 6. Conclusions and Future Work

In this paper, a balanced scheme between the robustness and real-time performance of a TTS is presented. A novel robust tracking algorithm combining the MS with template match (TM) has been proposed, which has a good robustness to target pose variation, partial occlusion, and a fast MS-based target tracking scheme is designed and implemented. The hardware-in-loop simulation shows that the image signal processing speed is >50 frame/s. The TTS presented in this paper utilized s common CCD camera to realize acquisition of images, but for some special applications infrared CCD sensors or heterogeneous sensors are used, so IR CCD or heterogeneous sensor-based fast target tracking techniques would be a future research direction.

## Figures and Tables

**Table 1.** The mainly performance of TDS642EVM.

**DSP**	**DSP Chip**	**TMXDM642**
	**Operating voltage**	**I/O:3.3 V Vcore:1.4 V**
	**Clock**	**600 MHz**
	**External bus clock**	**100 MHz**
Video In/Out	PAL/NTSC/SECAMS	
External Interface	RS232 UART	

**Table 2.** Performance of the 2D turntable.

Maximum Speed	10°/s
Rotation Range	Pitch: ±20°; Yaw: ±80°
Motor Type	Stepper Motor
Maximum Torque	2 Nm

**Table 3.** Comparison of CPU times for two cases.

	**Image 1**		**Image 2**	

	Number of iterations	CPU time	Number of iterations	CPU time
Fast MS	8	18.8 ms	7	14.2 ms
Standard MS	26	61.1 ms	28	60.8 ms

**Table 4.** TTS time-consuming statistical.

**Algorithm**	**Time**
Fast MS (10 iteration)	14.6 ms
TM	1.83 ms
Other	1.78 ms
Total	18.21 ms
